# Zyxin protects from hypertension-induced cardiac dysfunction

**DOI:** 10.1007/s00018-022-04133-4

**Published:** 2022-01-24

**Authors:** Jaafar Al-Hasani, Carla Sens-Albert, Subhajit Ghosh, Felix A. Trogisch, Taslima Nahar, Prisca A. P. Friede, Jan-Christian Reil, Markus Hecker

**Affiliations:** 1 Department of Cardiovascular Physiology, Heidelberg University, Im Neuenheimer Feld 326, 69120 Heidelberg, Germany; 2 Division of Cardiovascular Physiology, European Center for Angioscience, Heidelberg University, 68167 Mannheim, Germany; 3 Medical Clinic II, University Heart Center Lübeck, University Hospital Schleswig-Holstein, 23538 Lübeck, Germany

**Keywords:** Zyxin, Endothelial cells, Mechanotransduction, Hypertension, Left ventricular hypertrophy, Cardiac fibrosis

## Abstract

Arterial hypertension causes left ventricular hypertrophy leading to dilated cardiomyopathy. Following compensatory cardiomyocyte hypertrophy, cardiac dysfunction develops due to loss of cardiomyocytes preceded or paralleled by cardiac fibrosis. Zyxin acts as a mechanotransducer in vascular cells that may promote cardiomyocyte survival. Here, we analyzed cardiac function during experimental hypertension in zyxin knockout (KO) mice. In zyxin KO mice, made hypertensive by way of deoxycorticosterone acetate (DOCA)-salt treatment telemetry recording showed an attenuated rise in systolic blood pressure. Echocardiography indicated a systolic dysfunction, and isolated working heart measurements showed a decrease in systolic elastance. Hearts from hypertensive zyxin KO mice revealed increased apoptosis, fibrosis and an upregulation of active focal adhesion kinase as well as of integrins α5 and β1. Both interstitial and perivascular fibrosis were even more pronounced in zyxin KO mice exposed to angiotensin II instead of DOCA-salt. Stretched microvascular endothelial cells may release collagen 1α2 and TGF-β, which is characteristic for the transition to an intermediate mesenchymal phenotype, and thus spur the transformation of cardiac fibroblasts to myofibroblasts resulting in excessive scar tissue formation in the heart of hypertensive zyxin KO mice. While zyxin KO mice per se do not reveal a cardiac phenotype, this is unmasked upon induction of hypertension and owing to enhanced cardiomyocyte apoptosis and excessive fibrosis causes cardiac dysfunction. Zyxin may thus be important for the maintenance of cardiac function in spite of hypertension.

**Supplementary Information:**

The online version contains supplementary material available at 10.1007/s00018-022-04133-4.

## Introduction

Arterial hypertension is the primary risk factor for disease related deaths worldwide and tightly linked to cardiovascular disease. Arterial hypertension is an enigma, devoid of any obvious clinical signs but once established causes pressure overload in the heart, namely in the left ventricle, which consequently leads to a chronic increase in wall stress. Because of their inability to reenter the cell cycle, cardiomyocytes in the left ventricle respond with an enlargement that causes an increase in wall thickness hence normalizing wall stress [1]. Initially thought to be compensatory, this left ventricular (LV) hypertrophy is considered to be maladaptive as it impairs fractional shortening (FS) of the cardiomyocytes and thus may lead to systolic cardiac dysfunction and eventually heart failure [2].

In hypertension-induced cardiac fibrosis, the source of extracellular matrix (ECM) is controversially discussed. Cardiac fibroblasts in the myocardium can differentiate into more mobile and contractile myofibroblasts that harbor a greater synthetic ability to produce ECM proteins. Myofibroblasts are only appearing following cardiac injury whereas the healthy myocardium is devoid of these cells [3]. The major part of cardiac tissue is constituted by endothelial cells with 60% of non-myocytes in the heart [4]. Recently, the role of endothelial cells in the development of cardiac fibrosis increasingly came into focus as they seem to undergo a phenotypic switch, designated endothelial-to-mesenchymal transition (EndMT), which may give rise to excessive accumulation of myofibroblasts in the myocardium. Transforming growth factor-β (TGF-β), a key factor in inflammation, ECM remodeling and fibrosis, is thought to play an important role in EndMT [5]. Moreover, it has been shown that the collagen crosslinking enzyme lysyl oxidase (LOX) is essential for the development of cardiac fibrosis and mechanical dysfunction in different pathological situations [6]. Increased synthesis and accumulation of ECM proteins to replace cardiomyocytes and/or interrupt cardiomyocyte–cardiomyocyte interactions results in myocardial stiffening with a distorted ECM architecture and an overall impairment of cardiac function [7]. Furthermore, cardiac fibrosis disrupts the coordination of myocardial excitation–contraction coupling in both systole and diastole, thus facilitating arrhythmias [8].

Intramural cells sense their local chemo-mechanical environment and remodel the ECM to provide suitable compliance and sufficient strength [9]. Cortical actin networks are mechanically linked through integrin-containing, multi-protein structures as focal adhesions to the ECM, facilitating communication between the ECM and the cytoskeleton [10]. Costameres, the cardiomyocyte counterpart of focal adhesions in vascular cells, also play an important role in this bidirectional mechanotransduction [11]. The LIM domain protein zyxin, a component of focal adhesions, may be important for cardiomyocyte survival [12]. Zyxin regulates actin polymerization [13] and in response to cyclic stretch, as an in vitro surrogate for arterial hypertension, translocates to the nucleus of vascular cells. There it may regulate the expression of numerous mechanosensitive genes and thus maintain stretch-stimulated endothelial and smooth muscle cells in a quiescent state [14–16]. Whether zyxin plays a similar role in cardiomyocytes, cardiac fibroblasts or microvascular endothelial cells, thereby exerting a possible cardioprotective effect, is not known. Therefore, the aim of this work was to study the consequences of the global genetic loss of zyxin for cardiac function in two different mouse models of experimentally induced hypertension, the deoxycorticosterone acetate (DOCA) /high-salt (DOCA-salt) induced hypervolemia and the chronic exposure to angiotensin (Ang) II induced model of hypertension [17].

## Methods

### Animal experiments

All studies were carried out in strict accordance with regulations in Germany regarding the use of laboratory animals and were approved by the appropriate regulatory body of the State of Baden-Württemberg (Regional Council Karlsruhe, approval numbers: G-60/15, G-101/09 and G-217/14). C57BL/6J wild type (WT) mice were purchased from Charles River laboratories and zyxin knockout (KO) mice were bred and housed in the Interfaculty Biomedical Facility of Heidelberg University. Experimental hypertension was induced in 6 and 12 months old mice by DOCA or Ang II treatment (for a full outline of the different groups of animals used refer to Suppl. Table 1). In the DOCA-salt model, arterial blood pressure was monitored using radio telemetry probes (DSI PA-C10, DSI) placed at the aortic arch through the left common carotid artery under isoflurane (1–2%, v/v) anesthesia. Baseline blood pressure was recorded 7–8 days after probe implantation followed by subcutaneous DOCA-salt pellet (50 mg, 21-day release, Innovative Research of America) implantation, performed under isoflurane (1–2%, v/v) anesthesia, and drinking water supplementation with 1% (w/v) NaCl [18, 19]. Blood pressure was then monitored continuously for the next 21 days. Ang II (1.44 mg/kg BW/day) or vehicle was applied via an osmotic mini pump (Alzet 1002, DURECT Corporation) placed in the paravertebral pocket [20] under isoflurane (1–2%, v/v) anesthesia, and blood pressure was measured using the CODA^®^ tail-cuff method (Kent Scientific) under basal conditions (day 0) and following Ang II treatment (days 7 and 14). For cardiac function analyses during isoflurane (1–2%, v/v) anesthesia, a VisualSonics Vevo 2100 imaging system (FUJIFILM VisualSonics) equipped with a MS-550D transducer (22–55 MHz) was used. Morphology and function of the heart were analyzed in the parasternal long and short axis (PSLAX and SAX, respectively) view using the VEVO LAB software with VEVO strain extension. At the end of the experiment, anmials were left anesthetized after the last ultrasound recording, and euthanized via cervical dislocation for tissue collection. Serum levels of cystatin C were assayed by ELISA (Biovendor) following the manufacturer’s instructions.

### Working heart experiments

Working heart experiments were performed as described by Reil et al. [21]. Briefly, hearts were isolated, and the aortae were cannulated with a steel cannula connected to a heated preload column, to achieve a working temperature of 37 °C, and sewed in by a suture. To record LV systolic function, a high-fidelity conductance catheter (Millar, 1.4 F SPR-839) was inserted to the LV cavity by apical puncture. End-systolic pressure–volume relationship and slope end-systolic ventricular elastance were assessed by a sudden increase in afterload pressure from 90 to 150 mmHg through keeping the preload pressure constant at 5 mmHg. LV ejection fraction and stroke volume were also measured.

### Tissue preparation and staining

Excised perfused hearts were fixed in 4% *p*-formaldehyde followed by serial dehydration, and then embedded in paraffin. Five µm heart sections were immobilized on glass slides and stained with Masson’s trichrome. Sections were analyzed by light microscopy with ImageJ. For immunostaining, paraffin sections were deparaffinized and rehydrated. Apoptotic cells were detected by TUNEL staining with an in situ cell death detection kit (Roche) according to the manufacturer’s instructions.

### Isolation of primary cardiomyocytes and cardiac fibroblasts

Neonatal cardiomyocytes and cardiac fibroblasts were isolated from hearts of 1–2 days old mice by repeated digestion with DNase and collagenase. The cell suspension was transferred to a Petri dish and fibroblasts were allowed to adhere for 1 h. Cardiomyocytes remained in the supernatant, which were then either seeded on glass cover slips for immunofluorescence analysis or, for RNA isolation, on a plastic surface coated with 1% laminin solution and cultivated with DMEM F-12 Ham (Sigma-Aldrich) including 5% horse serum, 2 mmol/l l-glutamine and antibiotics. Neonatal cardiac fibroblasts were grown in DMEM medium containing 15% FBS and antibiotics. For stimulation experiments, cells were allowed to settle down, serum depleted overnight and stimulated for 24 h with 10 ng/ml recombinant TGF-β1 (R&D Systems) before harvesting.

### Cell lines and stretch assay

CI-muMECs (murine microvascular endothelial cells; #INS-CI-1004, InSCREENeX) were cultured in gelatin-coated culture flasks (Thermofisher Scientific) in high-glucose DMEM containing 20% FBS, 0.5% endothelial cell growth supplement from bovine neural tissue, 1% sodium pyruvate, 1% non-essential amino acids and antibiotics (all Sigma-Aldrich). For experiments, cells were plated in collagen type I BioFlex plates (Flexcell^®^ International) and transfected with control or zyxin siRNA (Qiagen) using MATra siRNA reagent (IBA) according to the manufacturer’s instructions. Following 72 h of culture, transfected cells were either exposed to cyclic stretch (15% elongation, 0.5 Hz) using a Flexcell^®^ FX-5000™ Tension System or incubated under static conditions for 6 h. Cells were then harvested and processed for protein analysis or RNA isolation.

### RNA isolation, cDNA synthesis and quantitative real time PCR

Excised heart tissue was stored in RNAlater™ stabilization reagent (Thermofisher Scientific) or homogenized in lysis solution using the gentleMACS™ dissociator (Miltenyi Biotec). For RNA isolation, the RNeasy^®^ mini kit (Qiagen) was used, and RNA was transcribed into cDNA using the Sensiscript^®^ reverse transcription kit (Qiagen). Real time PCR was performed using the QuantiTect SYBR Green^®^ kit (Qiagen). Expression levels were normalized to the reference gene *Rpl32*. Primer sequences and annealing temperatures are listed in Suppl. Table 2, and the conditions used for quantitative real-time PCR analysis are listed in Suppl. Table 3. To assess changes in pro-fibrotic gene expression an RT^2^ Profiler PCR Array Mouse Fibrosis (PAMM-120ZA, Qiagen) was used according to the manufacturer’s instructions and analyzed using an Applied Biosystems model 7500 real-time PCR cycler.

### Protein isolation and immunoblotting

Tissue samples were snap frozen and stored at − 80 °C or lysed in RIPA-buffer containing protease inhibitors and analyzed by immunoblotting. Primary antibodies used in this study were rabbit anti-zyxin (B71, provided by M. Beckerle, Huntsman Cancer Institute), anti-α-tubulin (#2144, Cell Signaling), anti-α-actinin (#A7811, Sigma-Aldrich), anti-FAK (#3285, Cell Signaling), anti-pFAK (Y^397^) (#44-624G, Thermo Fisher Scientific), anti-AKT (#9272, Cell Signaling), anti-pAKT (Ser^473^) (#9271, Cell Signaling), anti-Integrin α5 (#4705, Cell Signaling), anti-Integrin β1 (#9699, Cell Signaling), anti-Col1α2 (#sc-166865, Santa Cruz Biotechnology) and anti-LOX (sc-373995, Santa Cruz Biotechnology). HRP-conjugated anti-mouse (#4416, Sigma-Aldrich) and anti-rabbit (#6154, Sigma-Aldrich) antibodies were used as secondary antibodies and chemiluminescence was detected with the ImageQuant LAS mini system (GE Healthcare). Densitometric analysis was done with ImageJ.

### Statistical analysis

For data analysis, GraphPad Prism 7 (GraphPad) was used. Results are presented as means ± SEM. For analyzing differences between two individual experimental groups, unpaired Student’s *t* test was used with *p* < 0.05 considered statistically significant. Differences among pre and post treatment in experimental groups were first tested for normality using D'Agostino–Pearson omnibus test, and subsequently analyzed by two-way ANOVA followed by a Sidak's test for multiple comparisons, with a probability value of *p* < 0.05 considered statistically significant.

## Results

### Loss of zyxin induces cardiomyocyte apoptosis but has no effect on cardiomyocyte hypertrophy

**Fig. 1:**
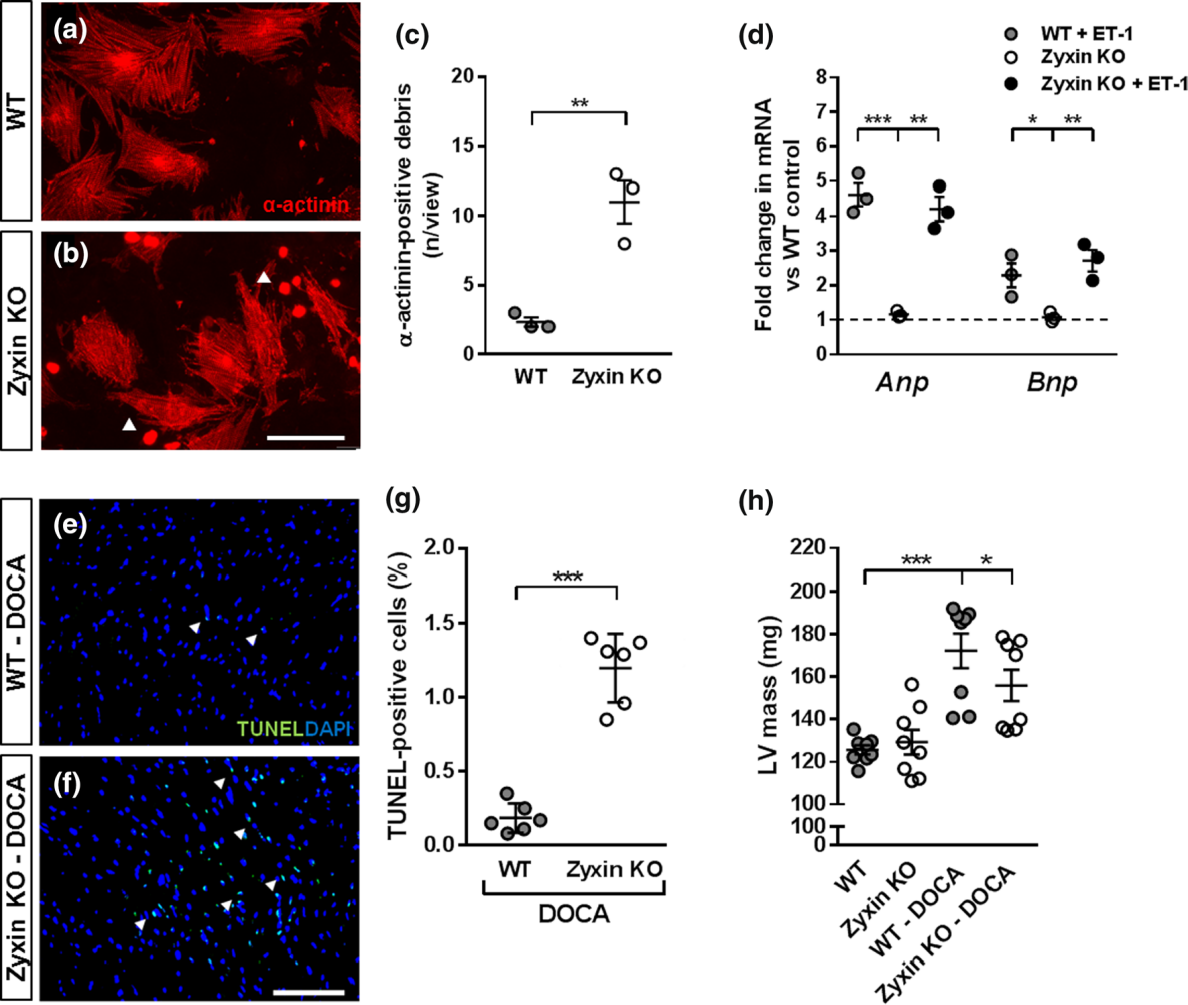
Apoptosis of primary cardiomyocytes and cardiac hypertrophy in 12-month old zyxin KO mice. **a**, **b** Staining for α-actinin in neonatal cardiomyocytes isolated from WT and zyxin KO mice, respectively (scale: 100 µm). **c** Quantification of α-actinin positive cellular debris (*n* = 3). **d** Relative expression levels of Anp and Bnp in WT and zyxin KO cardiomyocytes at baseline and following exposure to 100 nmol/l ET-1 for 24 h (*n* = 3). **e**, **f** TUNEL staining in LV tissue sections of DOCA-salt treated WT and zyxin KO mice (scale: 50 µm). **g** Quantification of the percentage of TUNEL-positive nuclei (*n* = 6). **h** LV mass analyzed by ultrasound imaging showing WT and zyxin KO mice under basal and DOCA-treated conditions (*n* = 8). *p < 0.05, **p < 0.01, ***p < 0.001

Neonatal cardiomyocytes isolated from WT or zyxin KO mice were serum-depleted for 24 h, fixed and stained for α-actinin. Zyxin-deficient cardiomyocytes consistently displayed a loss of viability as depicted by α-actinin-positive cell debris when compared to the characteristic z-line staining in WT cardiomyocytes (Fig. 1a–c). Exposing the neonatal cardiomyocytes to endothelin-1 (ET-1, 100 nmol/l) for 24 h, upregulated *Anp* and *Bnp* mRNA in both WT and zyxin-deficient cardiomyocytes, indicative of switching on the fetal gene program (Fig. 1d). The extent of apoptosis was analyzed by the number of TUNEL-positive nuclei detected in cardiac tissue sections, revealing a higher number of apoptotic cells in the hearts of DOCA-salt treated zyxin KO mice than in their WT counterparts (Fig. 1e–g). To compare the degree of hypertrophy, LV mass was determined by high-resolution echocardiography. As shown in Fig. 1h, there was a rise by 37% in WT and by 21% in 12-month old zyxin KO mice upon 21-day DOCA-salt treatment.

### Cardiac systolic dysfunction in hypertensive zyxin KO mice

**Fig. 2:**
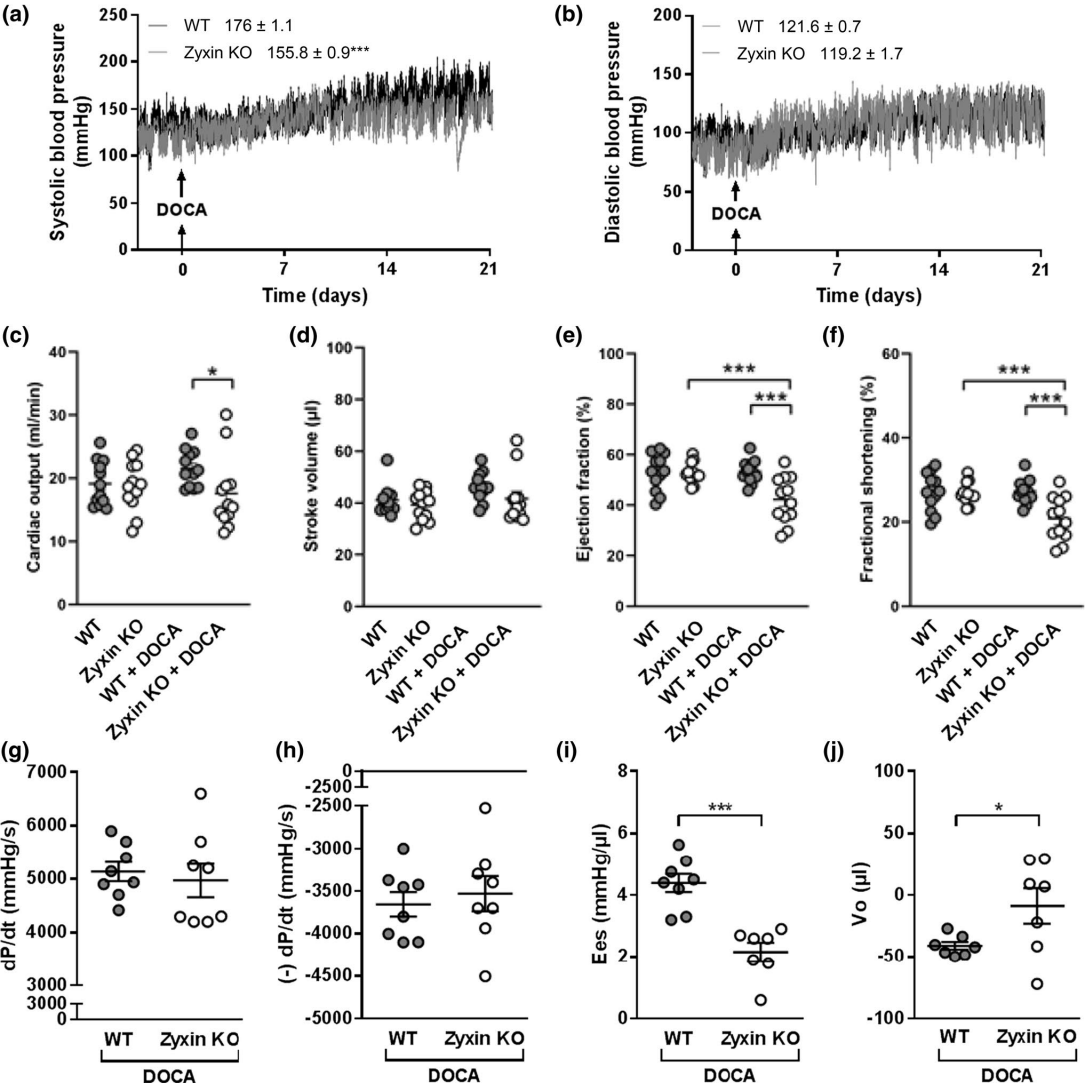
Cardiac systolic dysfunction in 12-month old DOCA-salt treated hypertensive zyxin KO mice. **a**, **b** Extent of systolic (**a**) and diastolic (**b**) blood pressure elevation in WT and zyxin KO mice after DOCA-salt treatment. The mean active blood pressure levels ± SEM are given in the graph legend (*n* = 8). **c**–**f** Echocardiographic analysis for cardiac output (**c**), stroke volume (**d**), ejection fraction (**e**) and fractional shortening (**f**) in DOCA-salt treated WT and zyxin KO mice (*n* = 8). **g**–**j** Peak positive value of the rate of developed LV pressure (d*P*/d*t*) (**g**), peak negative value of the rate of developed LV pressure (− d*P*/d*t*) (**h**), end-systolic elastance (Ees) (**i**) and end-diastolic pressure volume relationship (Vo) (**j**) in working hearts from DOCA-salt treated WT and zyxin KO mice (*n* = 7–8). *p < 0.05, **p < 0.01, ***p < 0.001

While blood pressure was comparable in 12-month old WT and zyxin KO mice at baseline, continuous radio telemetry recording revealed a similar increase in diastolic blood pressure in both mouse lines upon DOCA-salt treatment (Fig. 2b); however, systolic blood pressure was less elevated in zyxin KO than in WT mice (Fig. 2a). Cardiac function was analyzed by echocardiography in both groups, and no differences at baseline were found. After induction of hypertension, zyxin KO mice revealed a lower cardiac output (CO), ejection fraction (EF) and FS compared to WT mice, suggesting a chronic/end-stage hypertension-related systolic dysfunction (Fig. 2c, e, f). Although the DOCA-salt hypertension model is thought to be mainly neurogenic, it also affects the kidneys causing volume overload that may explain part of the rise in systolic blood pressure in this model [22]. To explore this possibility, serum concentrations of cystatin C, a marker of the glomerular filtration rate found at higher concentrations in kidney disease [23], were measured. However, cystatin C serum levels were not different between DOCA-salt treated WT and zyxin KO mice (Suppl. Fig. 1).

In 6-month old animals, DOCA-salt treatment consistently led to reduced cardiac function; EF and FS were significantly reduced, CO and SV also showed a mild yet not significant decrease (Suppl. Fig. 2d–g). Additionally, LV inner diameter (ID), mass and volume were increased (Suppl. Fig. 2a–c), while LV anterior and posterior wall (LV AW and PW, respectively) thickness were not changed (data not shown). Blood pressure in the 6-month old zyxin KO animals was not different from WT controls (data not shown).

To analyze cardiac parameters in detail, working heart measurements were performed using hearts isolated from 12-month old DOCA-salt treated animals. Zyxin-deficient mouse hearts showed decreased contractility and increased stiffness, as indicated by a reduced LV end-systolic elastance and an enhanced LV end**-**diastolic pressure volume relationship (Fig. 2i, j). Other parameters such as peak positive (d*P*/d*t*) and negative rates (− d*P*/d*t*) of developed LV pressure were not different between hypertensive WT and zyxin KO mouse hearts (Fig. 2g, h).

### Hearts of hypertensive zyxin KO mice show prominent fibrosis

**Fig. 3:**
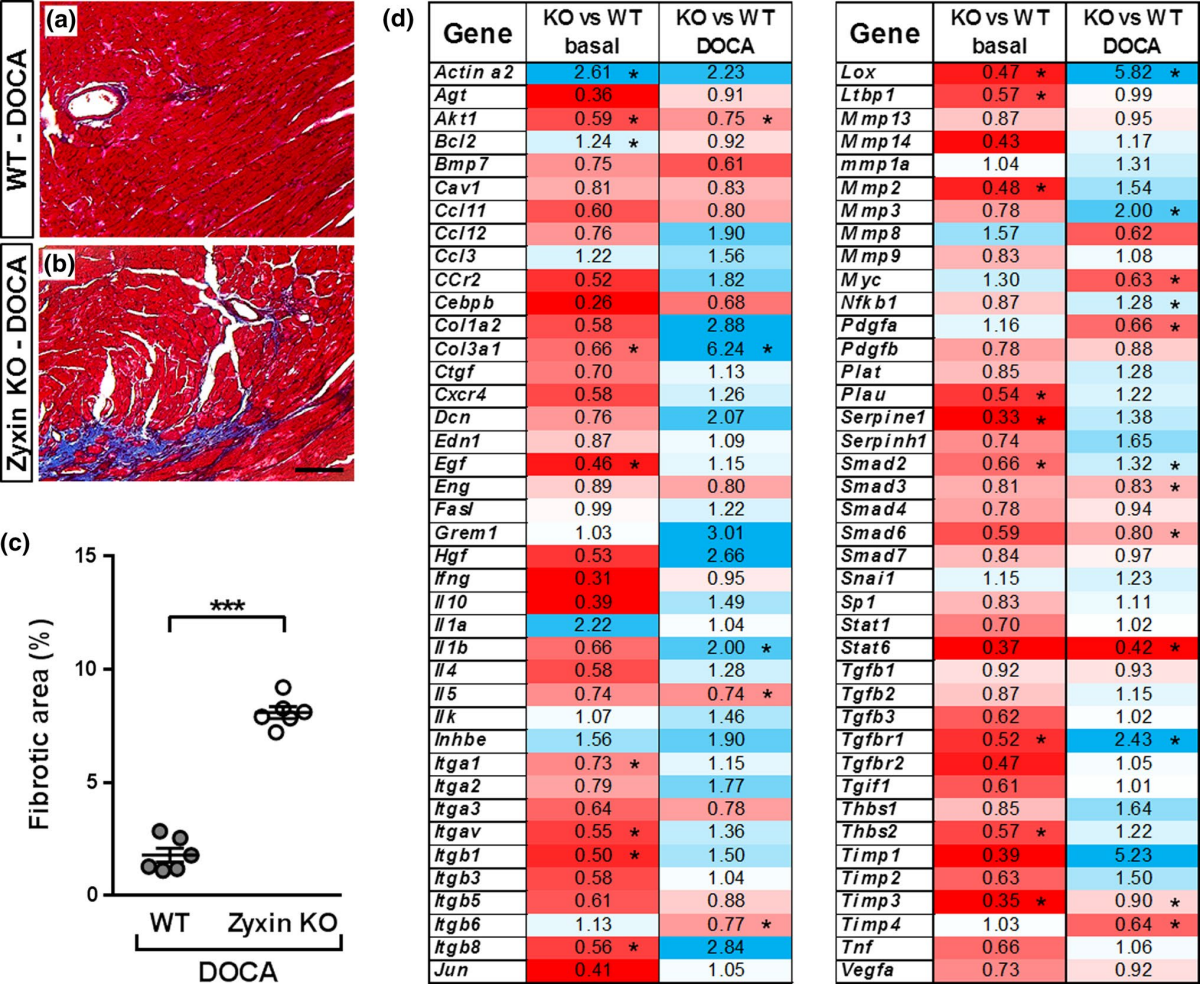
Cardiac fibrosis in 12-month old DOCA-salt treated zyxin KO mice. **a**, **b** Collagen deposition shown by Masson’s trichrome staining of hearts from DOCA-salt treated WT (**a**) and zyxin KO (**b**) mice (scale: 100 µm). **c** Quantification of the area of collagen deposition in the LV tissue sections (*n* = 6). **d** Representation of changes in mRNA expression of pro-fibrotic genes from heart tissue of WT and zyxin KO mice at baseline and following 10-day DOCA-salt treatment. Downregulated gene products are displayed in red, upregulated gene products are displayed in blue, while white color indicates no change. Significant changes are marked with asterisks (*n* = 3). *p < 0.05, ***p < 0.001

Transverse sections of hearts from DOCA-salt treated zyxin KO mice showed pronounced fibrosis as detected by Masson’s trichrome staining (Fig. 3a–c). To understand the origin of fibrosis, molecular changes were examined using a real time quantitative PCR-based profiler array to detect alterations in pro-fibrotic gene expression in hearts of zyxin KO as compared to WT mice at baseline and upon 10-day DOCA-salt treatment. Of the 84 analyzed gene products, *IL13*, *IL13ra2*, *Plg*, and *Serpina1a* were below the level of detection. At baseline, the majority of pro-fibrotic gene products were downregulated in hearts of zyxin KO mice. However, when made hypertensive the absence of zyxin favored a pronounced pro-fibrotic gene expression pattern (Fig. 3d).

### Ang II treated zyxin KO mice show reduced cardiac function

**Fig. 4:**
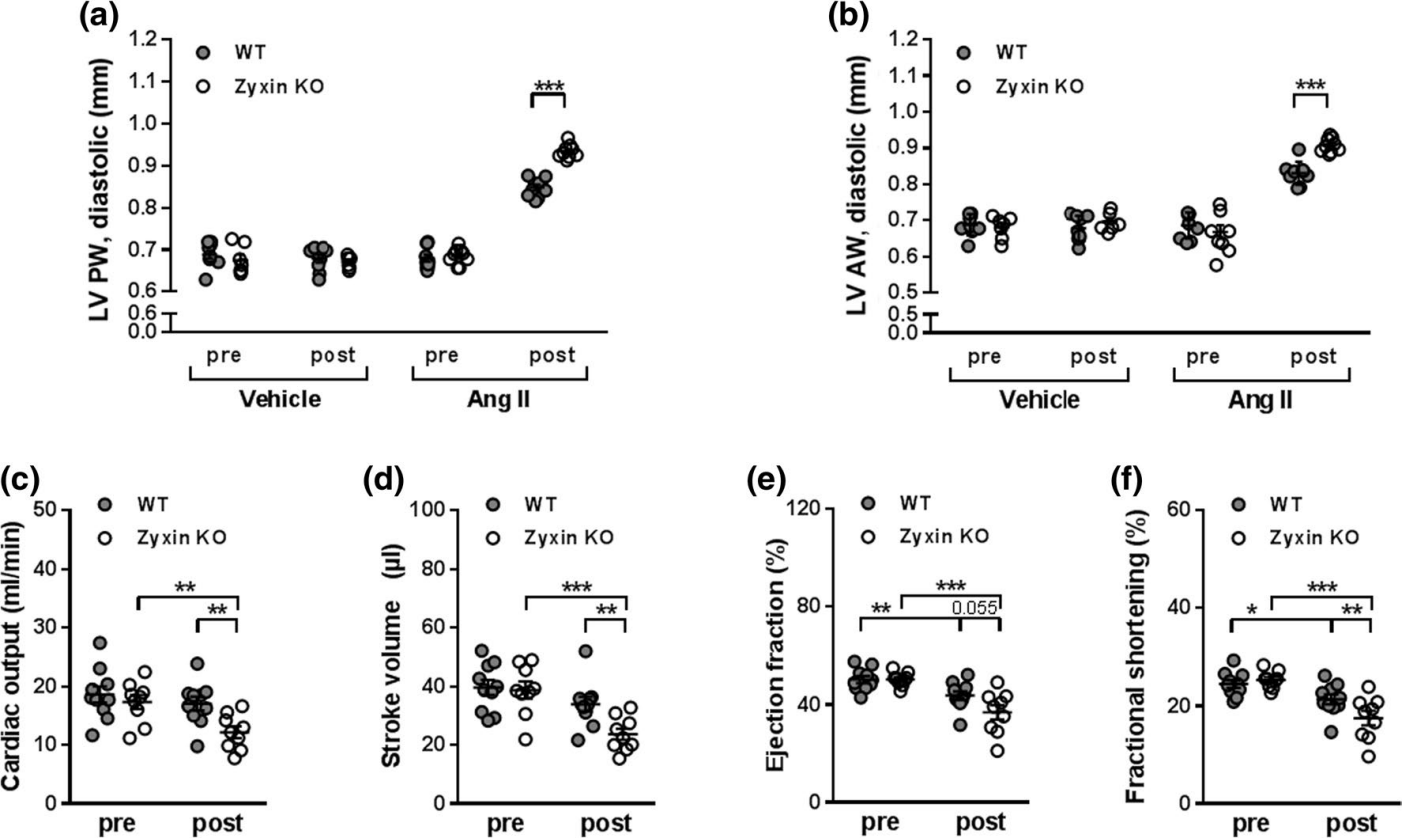
Cardiac systolic dysfunction in 12-month old Ang II treated WT and zyxin KO mice. **a**, **b** LV posterior (*n* = 9/7/9/7/10/9/10/9) (**a**) and anterior (*n* = 9/7/9/7/9/9/9/9) (**b**) wall thickness in WT and zyxin KO mice pre and post treatment with vehicle or Ang II. **c**–**f** Cardiac output (**c**), stroke volume (**d**), ejection fraction (**e**) and fractional shortening (**f**) in WT and zyxin KO mice before and after exposure to Ang II (*n* = 10/9/10/9). *p < 0.05, **p < 0.01, ***p < 0.001

Next, the consequences of a loss of zyxin were studied in a cardiac fibrosis model in which WT and zyxin KO mice were made hypertensive by continuous delivery of Ang II for 14 days [20]. Changes in blood pressure were monitored before and after treatment using non-invasive tail-cuff volume–pressure recording in awake animals. Systolic and diastolic blood pressure was elevated in both Ang II-treated WT and zyxin KO mice compared to their vehicle-treated counterparts (Suppl. Fig. 3a, b). This was significant in the wildtype but not in the KO mice. There was no difference in the relative increase of systolic or diastolic blood pressure between the 12-month old WT or zyxin KO mice (Suppl. Fig. 3c, d). Echocardiography revealed an increase in LV PW and AW thickness in both WT and zyxin KO mice in response to Ang II but this increase was more prominent in the latter (Fig. 4a, b). Moreover, Ang II treated mice presented with diminished cardiac function evidenced by a decline in CO, stroke volume (SV), EF and FS, which was more pronounced in the Ang II treated zyxin KO mice (Fig. 4c–f).

Similar findings were obtained with 6-month old zyxin KO mice. Thus, LV PW and AW thickness were more prominently increased in response to Ang II in these animals as compared to their WT counterparts. Interestingly, at this age, zyxin KO mice rather presented with an outward remodeling, as LV ID and volume were largely unaffected, while the WT animals showed more of an inward remodeling with a clear decline of both parameters. Cardiac function was also impaired in the 6-month old zyxin KO mice following Ang II treatment. Whereas both CO and SV were reduced, albeit not significantly greater than in the WT mice, the drop in both EF and FS was more pronounced in the zyxin KO mice (Suppl. Fig. 4). Such differences between WT and zyxin KO mice were not observed in 3-month old animals following treatment with Ang II (data not shown).

### Hypertensive zyxin KO mice show reduced cardiac tissue function and pronounced cardiac fibrosis

**Fig. 5:**
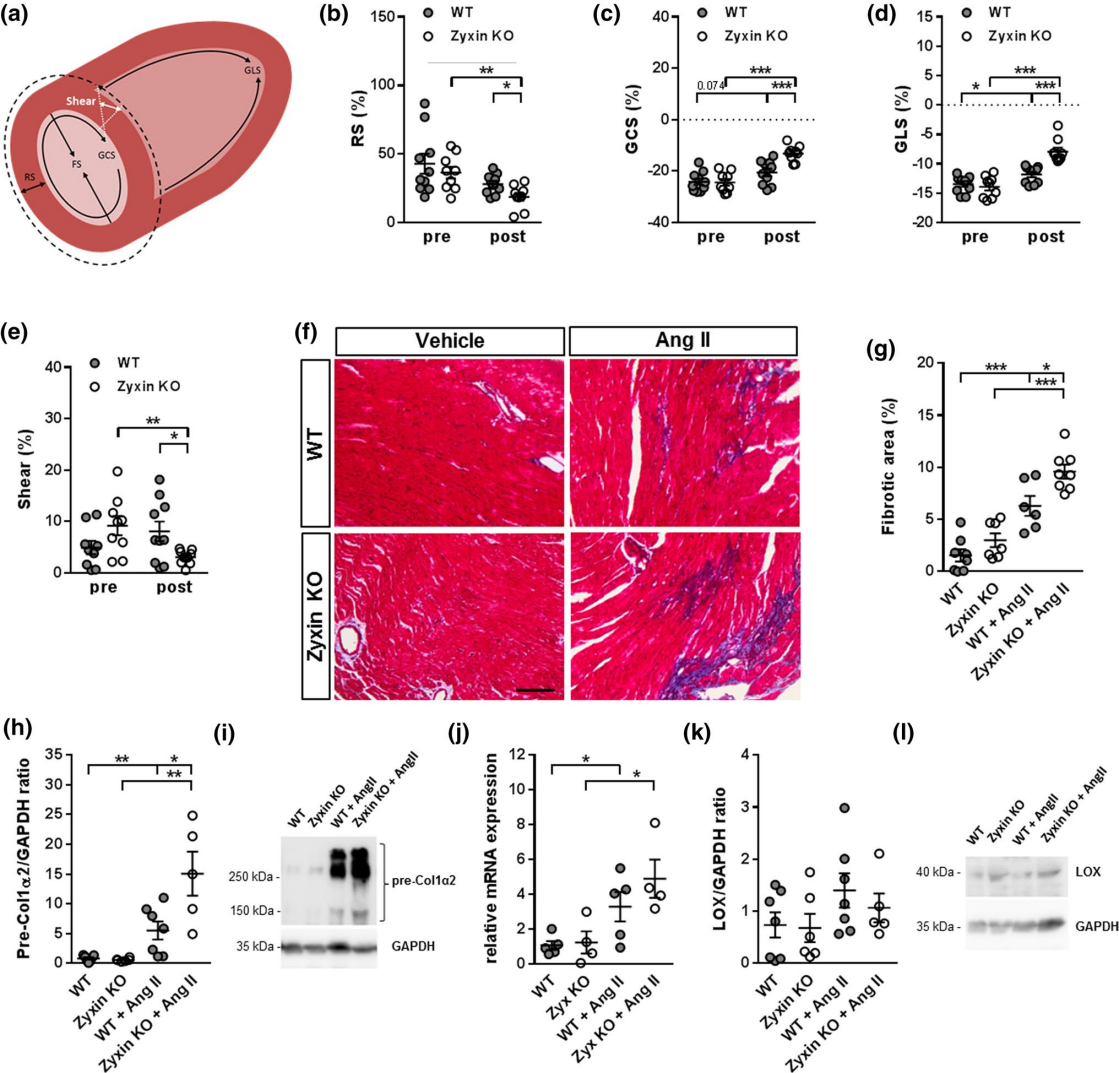
Cardiac stiffness and fibrosis in 12-month old Ang II-treated mice. **a** Schematic presentation of parameters to analyze cardiac contractility showing different strain and shear forces. **b**–**e** Echocardiographic evaluation of radial strain (RS, **b**), global circumferential strain (GCS, **c**), global longitudinal strain (GLS, **d**) and shear (**e**) in the left ventricle of WT and zyxin KO mice comparing only Ang II-treated animals pre and post treatment (*n* = 10/9/10/9). **f**, **g** Assessment of fibrosis development. Masson’s trichrome staining (**f**) and quantification (**g**) of collagen deposition in cardiac tissue sections of WT and zyxin KO mice treated with vehicle or Ang II (*n* = 8/7/6/8). (scale: 100 µm) **h**, **i** Quantification (**h**) and representative immunoblots (**i**) of pre-Col1α2 protein content normalized to GAPDH in hearts from control or Ang II treated WT or zyxin KO mice (*n* = 7/6/7/5). **j**–**l** Analysis of LOX expression. Relative *Lox* mRNA expression (**j**) in hearts from vehicle or Ang II treated WT or zyxin KO mice (*n* = 5/4/5/4). Quantification of LOX protein in heart lysates (**k**) of the four treatment groups (*n* = 7/6/7/5), and representative immunoblots (**l**). *p < 0.05, **p < 0.01, ***p < 0.001

Using speckle-tracking echocardiography tracing of myocardial deformation or strain for quantification of LV function [24], radial strain (RS), global circumferential strain (GCS) and global longitudinal strain (GLS) levels were evaluated. A reduction in all strain parameters in 12-month old WT and zyxin KO mice post Ang II treatment was observed, which in the zyxin-deficient animals was more pronounced than in their WT counterparts (Fig. 5a–d). The more severe functional impairment in the hypertensive zyxin KO mice was reflected by slower radial relaxation and contraction velocities (RRV and RCV, respectively) as well as by a decrease in both systolic and diastolic circumferential strain rate (CSR) (Suppl. Fig. 5a–d). Furthermore, a drop in shear movement of the myocardial tissue in Ang II treated zyxin KO mice was noted indicating a stiffening of the cardiac tissue due to potential fibrosis (Fig. 5e). Similar effects were observed in 6-month old Ang II-treated zyxin KO and WT mice (Suppl. Fig. 6).

Following Ang II treatment, an increased collagen deposition, as shown by Masson’s trichrome staining (Fig. 5f), was found in transverse sections of both WT and zyxin KO mouse hearts, and once again was more pronounced in the latter (Fig. 5g). Immunoblotting confirmed an elevation in collagen 1α2 protein in the heart of Ang II treated zyxin KO mice as compared to their WT counterparts (Fig. 5h, i). In addition, the Ang II treated zyxin KO mice revealed an increased *Lox* mRNA expression (Fig. 5j), similarly to the DOCA-salt treated zyxin KO mice (cf. Fig. 3d). However, lysyl oxidase (LOX) protein abundance was not altered in the hearts of the Ang II treated mice (Fig. 5k, l).

### Integrin expression and activation in hearts of hypertensive zyxin KO mice

Integrin expression and activation regulates the phosphorylation of focal adhesion kinase (FAK), a protein kinase that plays an important role in fibrosis [25]. Protein lysates from hearts of DOCA-salt treated mice were analyzed for the abundance of integrins α5 and β1 and active, phosphorylated FAK. Both integrins as well as active FAK were upregulated in hearts of zyxin KO mice (Suppl. Fig. 7a, b). In contrast, the phosphorylation of AKT was not different in DOCA-salt treated WT or zyxin KO mice.

### Origin of pro-fibrotic factors in hearts of zyxin KO mice

**Fig. 6:**
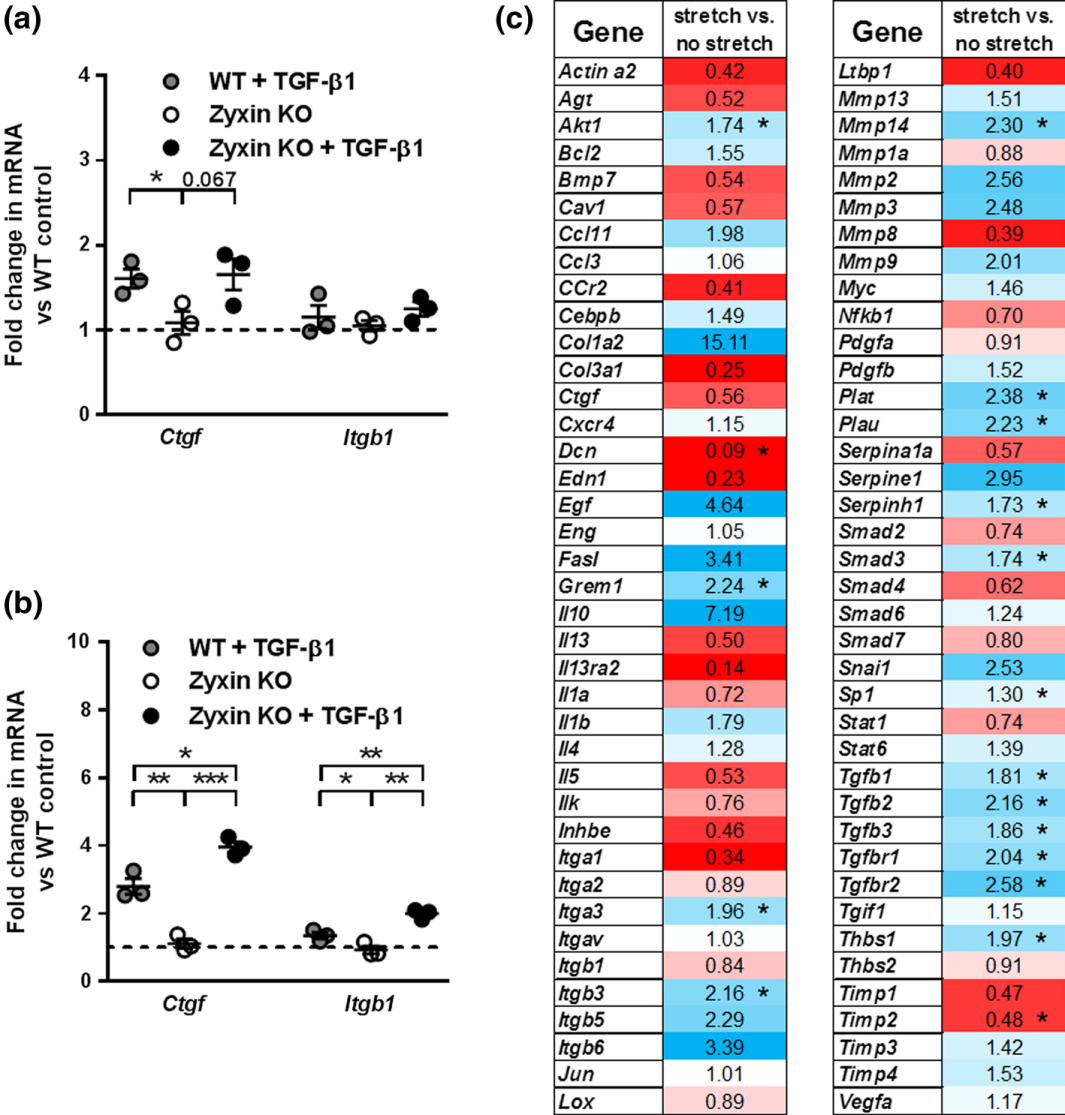
Origination of pro-fibrotic factors in hearts of zyxin KO mice. **a**, **b** Relative mRNA expression levels of *Ctgf* and *Itgb1* at baseline and following stimulation with TGF-β1 (10 ng/ml) in neonatal cardiomyocytes (**a**) and neonatal cardiac fibroblasts (**b**) (*n* = 3 each). **c** Heat map representation of changes in mRNA expression of pro-fibrotic genes analyzed by the RT^2^-profiler array in microvascular endothelial cells cultured under static conditions or exposed to cyclic stretch. Downregulated gene products are displayed in red, upregulated gene products are displayed in blue, while white color indicates no change. Significant changes are marked with asterisks (*n* = 3–6/3–6). *p < 0.05, **p < 0.01, ***p < 0.001

To identify the cell type responsible for the prominent cardiac fibrosis observed in hypertensive zyxin KO mice, cardiomyocytes and cardiac fibroblasts isolated from neonatal WT and zyxin KO mice were serum-depleted for 24 h and then stimulated with recombinant TGF-β1 for 24 h. TGF-β1 induced a comparable upregulation of *Ctgf* and *Itgb1* mRNA expression in neonatal cardiomyocytes from both WT and zyxin KO mice (Fig. 6a). In neonatal cardiac fibroblasts, TGF-β1 treatment increased *Ctgf* mRNA levels more prominently in cells isolated from zyxin KO mice (Fig. 6b). *Itgb1* mRNA expression was induced only in zyxin-deficient neonatal cardiac fibroblasts (Fig. 6b). As endothelial cells constitute the major portion of nonmyocyte cells in the mouse heart [4], and because zyxin seems to be strongly enriched both in macro- and microvascular endothelial cells of adult mice [14, 15] just as in humans (human protein atlas, https://www.proteinatlas.org/ENSG00000159840-ZYX/cell#rna, accessed on 23 June 2021), immortalized murine microvascular endothelial cells (CI-muMECs) were exposed to cyclic stretch in vitro to simulate the situation in the hypertensive mouse heart. In response to cyclic stretch, CI-muMECs upregulated different isoforms of TGF-β and their receptors, as well as *Akt1*, *Grem1*, *Itga3*, *Itgb3*, *Mmp14*, *Plat*, *Plau*, *Smad3*, *Sp1*, and *Thbs1*, while *Dcn* and *Timp2* were downregulated. Interestingly, *Col1a2* mRNA expression was strongly albeit not significantly increased in stretched CI-muMECs made zyxin-deficient via siRNA-mediated knockdown with a mean efficacy of 75%, suggesting that microvascular endothelial cells in the heart of zyxin KO mice could be a source of collagen as well (Fig. 6c). Out of the 84 genes in the array analyzed following knockdown of zyxin, *Ccl12*, *Hgf*, *Ifng*, *Itgb8*, *Plg* and *Tnf* mRNA levels were below the limit of detection and therefore are not displayed in Suppl. Fig. 8a, b. Of the remaining gene products analyzed only two revealed changes in expression of the corresponding gene. *Itga2* mRNA levels were significantly higher in stretched zyxin-deficient CI-muMECs, while cyclic stretch stimulated *Itgb6* mRNA expression solely in wildtype control CI-muMECs (Suppl. Fig. 8c, d).

## Discussion

In DOCA-salt induced hypertension, zyxin KO mice, in an age-dependent manner, revealed a systolic but not diastolic cardiac dysfunction accompanied by prominent cardiac fibrosis and upregulation of several pro-fibrotic gene products in the myocardium. This pro-fibrotic gene expression pattern was not only absent in hearts of zyxin KO mice at baseline but rather reflected an anti-fibrotic profile instead, pointing to an important maintenance function of zyxin under conditions of pressure overload. As DOCA-salt administration upregulates the renin-angiotensin system [26], we also studied Ang II-induced hypertension, which likewise in an age-dependent manner led to cardiac hypertrophy both in WT and zyxin KO mice paralleled by severe perivascular and interstitial fibrosis, which was particularly prominent in the hearts of the zyxin KO mice.

Interestingly, no such cardiac phenotype was observed in 3-month old zyxin KO mice at baseline or following induction of experimental hypertension with either Ang II or DOCA-salt. This phenotype declined further in terms of blood pressure control but not left ventricular function in preliminary experiments with 18-month old zyxin KO mice treated with DOCA-salt as compared to age-matched WT control animals (data not shown). A possible explanation for this finding is that, rather than being due to the loss of zyxin, it may be caused by an age-dependent decline of the zyxin homologue lipoma-preferred partner (LPP) in resistance-sized arteries, which determine total peripheral resistance (TPR) and thus blood pressure (data not shown). LPP is capable of restoring the contractile phenotype in zyxin-deficient vascular smooth muscle cells [27], so that its decline in the arteries of the 18-month old zyxin KO mice may add up to the cardiac dysfunction in these animals, hence aggravating their dysfunctional blood pressure response to the DOCA-salt treatment. Accordingly, 18-month old DOCA-salt treated zyxin KO mice reveal a significantly lower resistivity index, which is an indirect measure of TPR, than their WT counterparts (cf. Suppl. Fig. 9).

Cardiac fibrosis in hypertension is complex, raising the question which cell type, the cardiomyocytes, cardiac fibroblasts or endothelial cells, accounts for the increased fibrosis observed in the heart of the hypertensive zyxin KO mice. This profibrotic response may be initiated by cardiomyocyte loss and insufficient wound healing, as zyxin has been reported to protect cardiomyocytes from apoptosis by consolidating AKT [12]. Failing hearts of spontaneously hypertensive rats display a co-localized expression of collagen I in areas of cardiomyocyte degeneration [28]. In accordance, isolated cardiomyocytes from zyxin KO mice show enhanced apoptosis that may initiate deposition of ECM components. In the cardiac interstitium of spontaneously hypertensive rats and hypertensive patients, elevated levels of collagen lead to an increase in left ventricular stiffness [29, 30]. Our observations indicate that increased cardiac fibrosis leads to myocardial stiffening in hypertensive zyxin KO mice. Accordingly, analysis of myocardial deformation potential revealed a reduction in radial strain, myocardial shear, global circumferential strain and global longitudinal strain in Ang II treated zyxin KO mice. While deterioration in mycoardial shear suggests impairment in the force conductance from fiber shortening to wall thickening, it has also been reported that ventricular stiffness could be positively correlated with the degree of collagen cross-linking in human heart failure [31]. In this context, hearts from DOCA-salt treated zyxin KO mice showed elevated lysyl oxidase expression, a finding that was not observed in the hearts of these mice after long-term exposure to Ang II.

In the 6-month old DOCA-salt treated zyxin KO mice cardiac output and stroke volume were not significantly different from their WT counterparts while ejection fraction and fractional shortening were clearly reduced. However, the hearts of the zyxin KO mice showed signs of a moderate dilation (increased left ventricular internal diameter, increased left ventricular volume, increased left ventricular mass) that may have prevented the fall in cardiac output and stroke volume, which is clearly visible in the 12-month old zyxin KO mice made hypertensive with DOCA-salt. The degree of fibrosis in the hearts of these animals is much higher so that it can no longer be compensated and all four parameters of left ventricular cardiac function drop significantly as compared to the DOCA-salt treated WT mice.

ECM components can influence cardiac contractility by altering integrin signaling [32]. For example, overexpression of integrin αvβ1 stimulates expression of TGF-β1 while TGF-β1 conversely can induce expression of αvβ1 [33]. Moreover, integrin α5β1 has been reported to be elevated in cardiomyocytes from hypertrophic hearts and has been implicated in cardiac development [34–36]. In hypertensive zyxin KO hearts, there was an upregulation of integrins α5 and β1. This might be a downstream effect of increased TGF-β1 signaling via TGFβRI, which is more prominently expressed in zyxin KO hearts after DOCA-salt treatment. In hypertension, mechanical deformation is sensed by integrins and transduced through downstream signaling molecules like focal adhesion kinase, which was more active in the hearts of zyxin KO mice, as indicated by an increased phosphorylation at Y397 [37].

Myocardial stiffness in terms of fibrosis is attributed to changes in abundance and turnover of fibrillar collagens regulated by several growth factors such as Ang II, TGF-β1 and CTGF [7, 38]. Hearts from zyxin KO mice display no pro-fibrotic phenotype at baseline while this becomes quite prominent upon DOCA-salt induced hypertension, suggesting that cardiomyocyte death triggered accumulation of ECM components could be reinforced by the absence of zyxin. Whereas neonatal cardiomyocytes isolated from both WT and zyxin KO mice do not respond differently to stimulation with TGF-β1, neonatal cardiac fibroblasts isolated from zyxin KO mice seem to be more sensitive to TGF-β1 stimulation by upregulating CTGF and integrin β1 expression more prominently than their WT counterparts.

Increased myocardial TGF-β1 expression during cardiac hypertrophy and fibrosis has been observed in humans as well as in various animal models [39]. Treatment with Ang II has been reported to induce TGF-β1 expression in the myocardium facilitating the transformation of cardiac fibroblasts into myofibroblasts [7]. During fibrosis, matrix stiffness increases, hence raising the mechanical load on affected cells [40]. To mimic this pressure overload-dependent mechanical strain, we exposed microvascular endothelial cells to cyclic stretch. Interestingly, TGF-β isoforms and their corresponding receptors were expressed more prominently in response to this biomechanical stimulus in zyxin-deficient endothelial cells. TGF-β1 activation is, beside other mechanisms, regulated by biomechanical forces acting on the ECM and this is reinforced by its increased stiffness [41]. Moreover, TGF-β1 has been implicated in EndMT, and one study reported that 27–35% of fibroblasts in the fibrotic myocardium originate from endothelial cells [5]. In our hands, stretched microvascular endothelial cells increase collagen 1α2 expression, reflecting a phenotypical change associated with differentiation to an intermediate EndMT-like phenotype. Full transdifferentiation of microvascular endothelial cells to myofibroblasts in the left ventricle upon pressure overload as a primary source of fibrotic tissue in cardiac fibrosis is still a matter of debate [42]. However, in combination with an enhanced zyxin-dependent differentiation of fibroblasts to myofibroblasts, increased adoption of the EndMT-like phenotype by the zyxin-deficient microvascular endothelial cells may lead to the excessive interstitial fibrosis observed in the hypertensive zyxin KO mice.

As zyxin KO cardiomyocytes show a lower survival rate under basal conditions, hearts of zyxin KO mice may thus already be primed to develop hypertrophy induced by hypertension, which apparently is paralleled by pronounced fibrosis. The excessive accumulation of ECM proteins may be triggered by zyxin-deficient cardiac fibroblasts reacting more sensitively to TGF-β1. Once established, fibrotic tissue rigidifies the heart, thereby intensifying the enhanced mechanical load on all cells in the heart and on the ECM. Besides activating further TGF-β1 from the stiffening ECM, zyxin-deficient endothelial cells may produce more TGF-β isoforms, as we found in microvascular endothelial cells exposed to cyclic stretch. This may trigger a vicious cycle by again stimulating cardiac fibroblasts to differentiate to matrix producing myofibroblasts or by induction of the intermediate EndMT-like endothelial cell phenotype, resulting in excessive cardiac fibrosis in hypertensive zyxin KO mice. We therefore propose that zyxin, apart from its role as a mechanotransducer, maintains cardiac function in the face of a developing maladaptive response to hypertension.

**Fig. 7:**
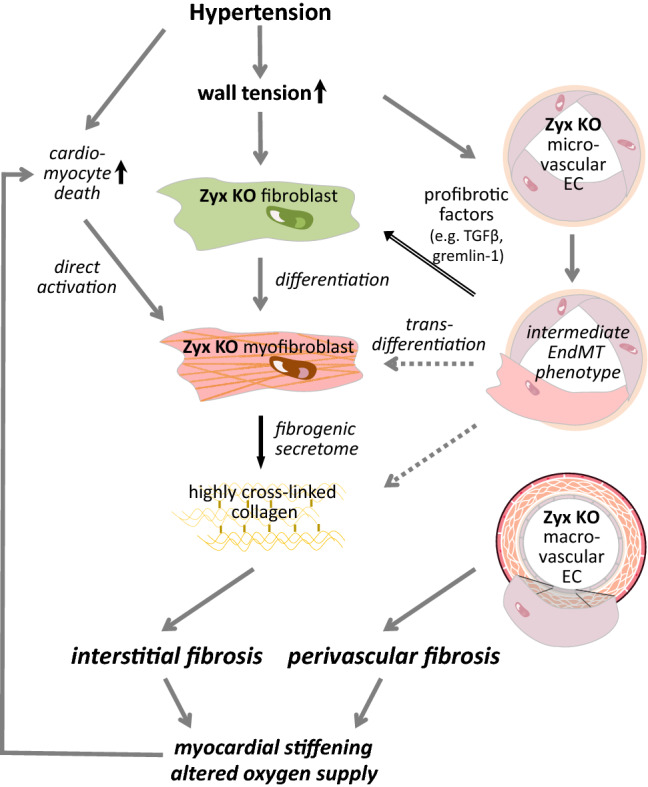
Graphical abstract summarizing the results of the research work. Experimental hypertension leading to a prolonged increase in wall tension hence stretch causes a differentiation of zyxin-deficient microvascular endothelial cells (EC) to an intermediate endothelial-to-mesenchymal transition (EndMT)-like phenotype that by releasing profibrotic factors spurs the differentiation of zyxin-deficient cardiac fibroblasts into myofibroblasts. The subsequent increased release of collagens and cross-linking enzymes from the activated myofibroblasts results in an enhanced interstitial fibrosis, which does not occur in the heart of hypertensive wildtype mice. In addition, stretched zyxin-deficient macrovascular EC release collagens and ECM-modifying enzymes causing perivascular fibrosis. Both effects are more pronounced when angiotensin II instead of DOCA-salt is used to elicit experimental hypertension because of the well-known profibrotic effects of angiotensin II. The resulting myocardial stiffening and altered oxygen supply exacerbates cardiomyocyte death, which by direct activation of the zyxin-deficient myofibroblasts reinforces interstitial cardiac fibrosis. Whether there is also a transdifferentiation of the microvascular EC adopting an intermediated EndMT phenotype into myofibroblasts remains to be investigated

In conclusion, the main mechanisms through which zyxin may protect the hypertensive mouse heart are summarized in Fig. 7.

## Supplementary Information

Below is the link to the electronic supplementary material.

Supplementary file1 (DOCX 2020 kb)

## Data Availability

Any material other than available from commercial sources described in this manuscript can be obtained from the authors upon written request.
