# Supplementary material for: Zyxin protects from hypertension-induced cardiac dysfunction
Source: Cell Mol Life Sci. 2022 Jan 24;79(2):93. doi: 10.1007/s00018-022-04133-4 (PMC8786748; doi:10.1007/s00018-022-04133-4)
Supplement: Supplementary file 1 — Supplementary file1 (DOCX 2020 kb) [file 18_2022_4133_MOESM1_ESM.docx]

# Supplemental Information


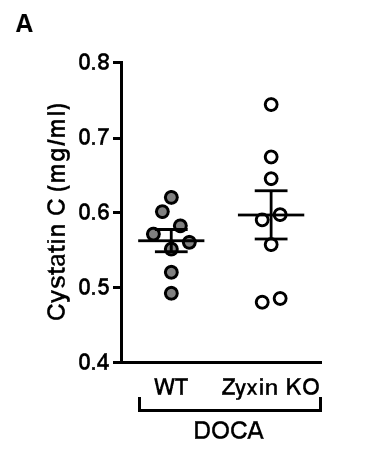


Suppl. Fig. 1 Cystatin C in 12-month old DOCA-salt treated mice.

Quantitative analysis of cystatin C concentrations in serum collected from 12-month old WT and zyxin KO animals at the end of DOCA-salt treatment (*n=8*).


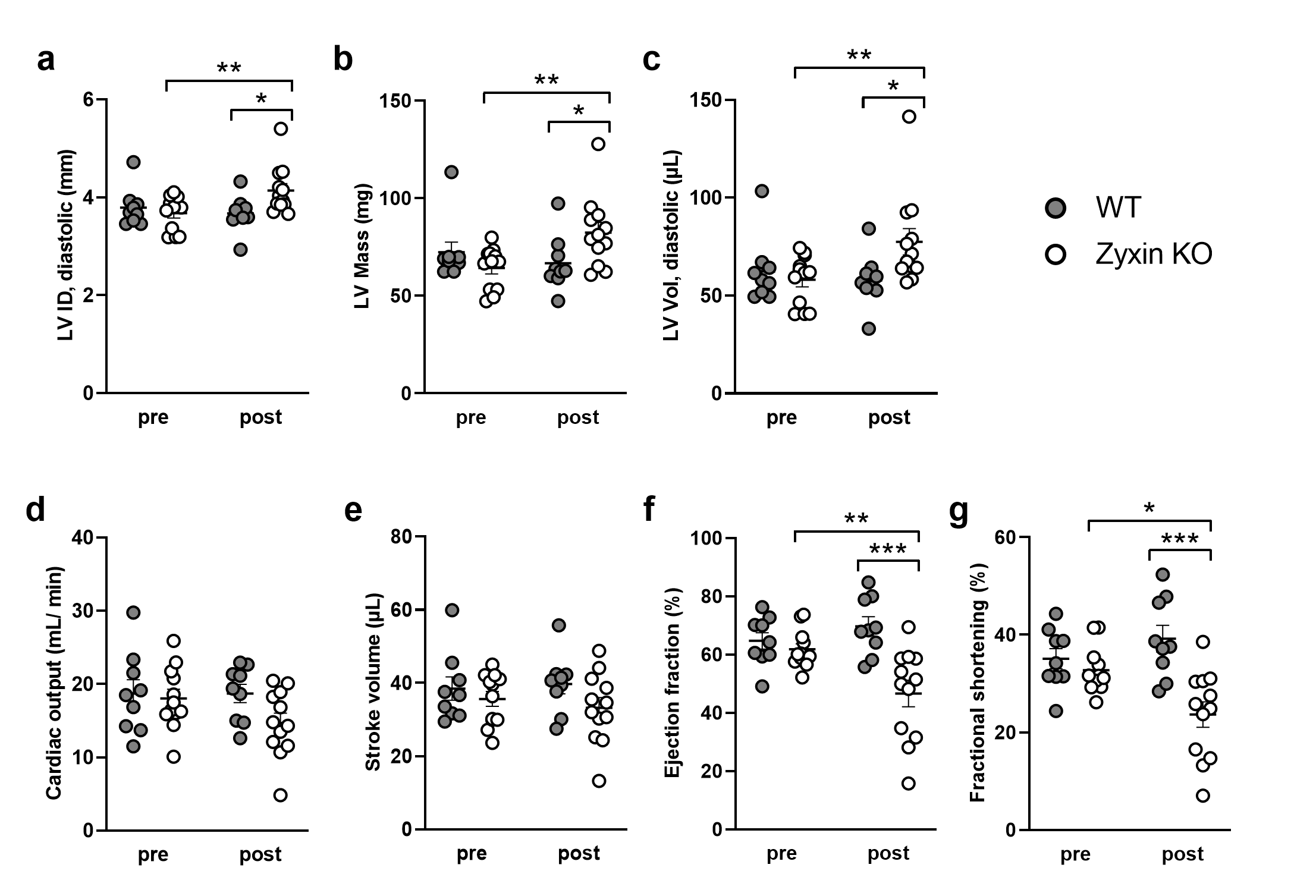


Suppl. Fig. 2 Cardiac systolic dysfunction in DOCA-salt treated 6-month old mice.

a-c Echocardiographic analysis for LV remodeling, comparing LV inner diameter (a), mass (b) and volume (c) from before and after treatment. d-g Analysis of functional systolic parameters cardiac output (d), stroke volume (e), ejection fraction (f), and fractional shortening (g) (*n=9/12*). *p<0.05, **p<0.01, ***p<0.001


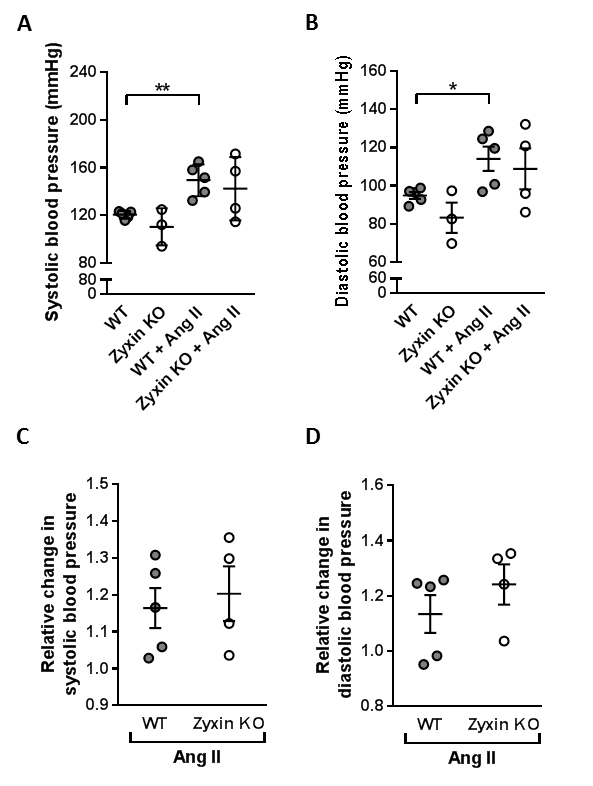


a

b

c

d

**Diastolic blood pressure (mmHg)**

Suppl. Fig. 3 Blood pressure measurements in vehicle or Ang II treated 12-month old WT and zyxin KO mice.

a, b Non-invasive tail cuff-based measurements of systolic (a) and diastolic (b) blood pressure on day 14 (*n=5/3/5/4*). c, d Relative changes in systolic (c) and diastolic (d) blood pressure between Ang II WT and zyxin KO mice (*n=5/4*). *p<0.05, **p<0.01


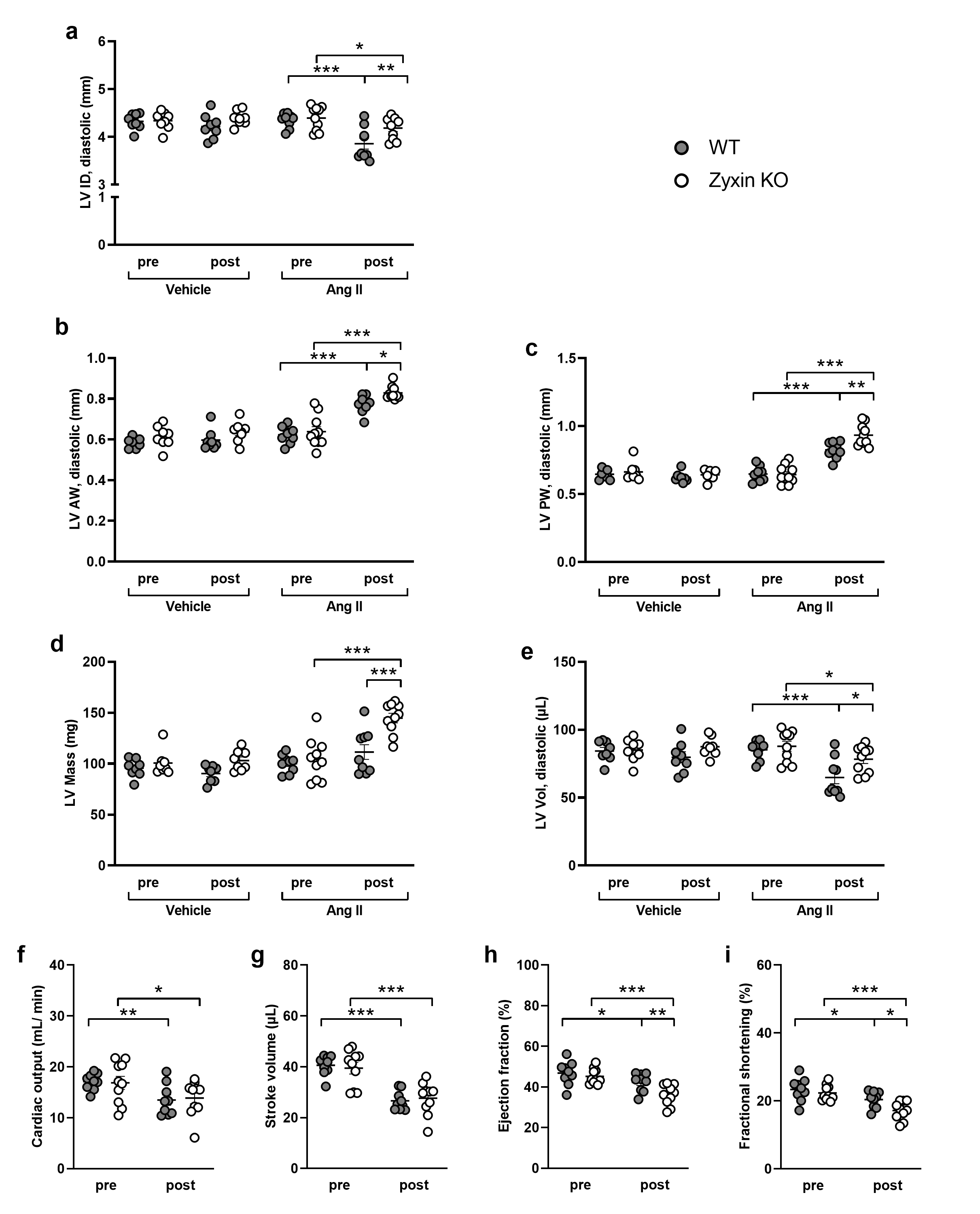


Suppl. Fig. 4 Echocardiographic evaluation of cardiac remodeling and function in Ang II treated 6-month old mice.

**a-e** Echocardiographic analysis for LV remodeling, comparing LV inner diameter (a), anterior (b) and posterior (c) wall thickness, mass (d) and volume (e) from vehicle and Ang II treated WT and zyxin KO animals (n= 8/8/9/10). **f-i** Analysis of functional systolic parameters cardiac output (f), stroke volume (g), ejection fraction (h), and fractional shortening (i), comparing only Ang II-treated mice (n=9/10). *p<0.05, **p<0.01, ***p<0.001


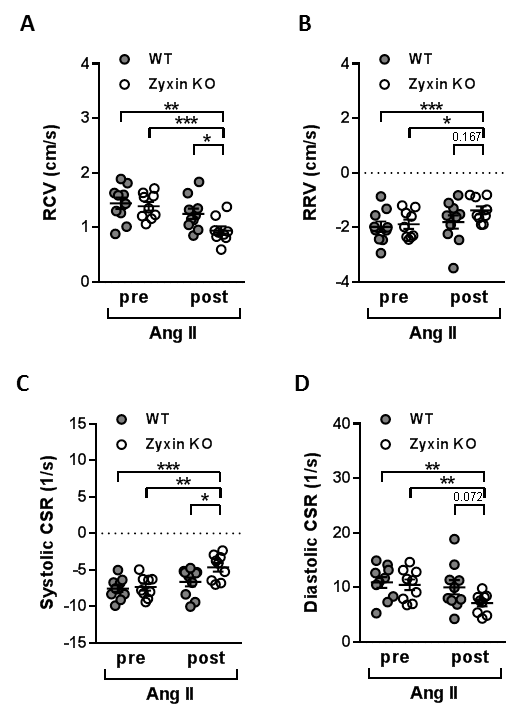


a

b

c

d

Suppl. Fig. 5 Echocardiographic evaluation of systolic and diastolic function in Ang II treated 12-month old mice.

a Analysis of radial contraction velocity, RCV. b Evaluation of radial relaxation velocity, RRV (*n=10/9/10/9*). c, d Systolic (c) and diastolic (d) circumferential strain rate, CSR, of zyxin KO and WT mice pre and post Ang II treatment (*n=10/9/10/9*). *p<0.05, **p<0.01, ***p<0.001


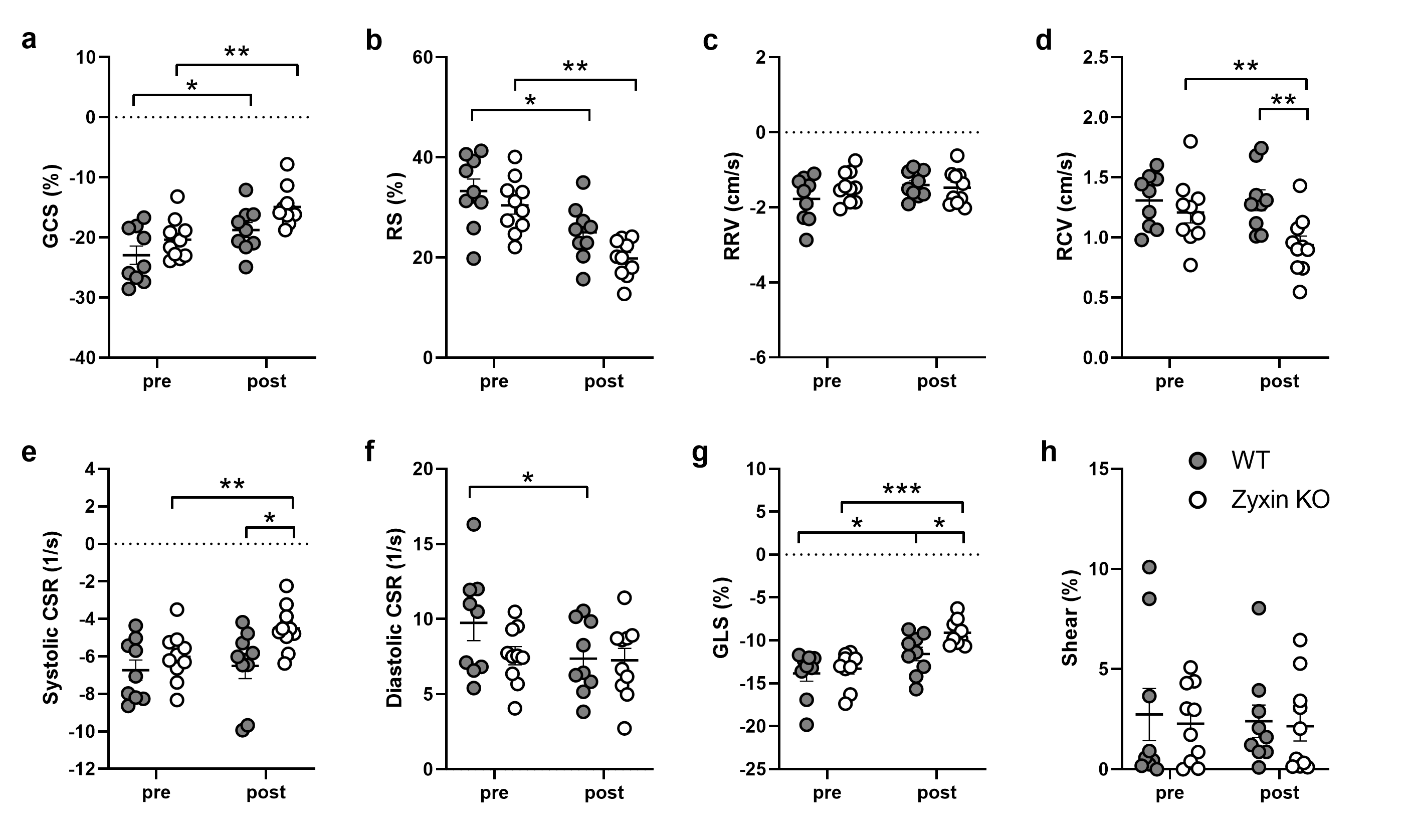


Suppl. Fig. 6 Echocardiographic evaluation of systolic and diastolic function in Ang II treated 6-month old mice.

Analysis of global circumferential strain, GCS (a), radial strain, RS (b), radial relaxation velocity, RRV (c), radial contraction velocity, RCV (d), systolic (e) and diastolic (f) circumferential strain rate, CSR, as well as global longitudinal strain, GLS (g) and shear (h) in 6-month old zyxin KO and WT mice pre and post Ang II treatment (*n=9/10*). *p<0.05, **p<0.01, ***p<0.001


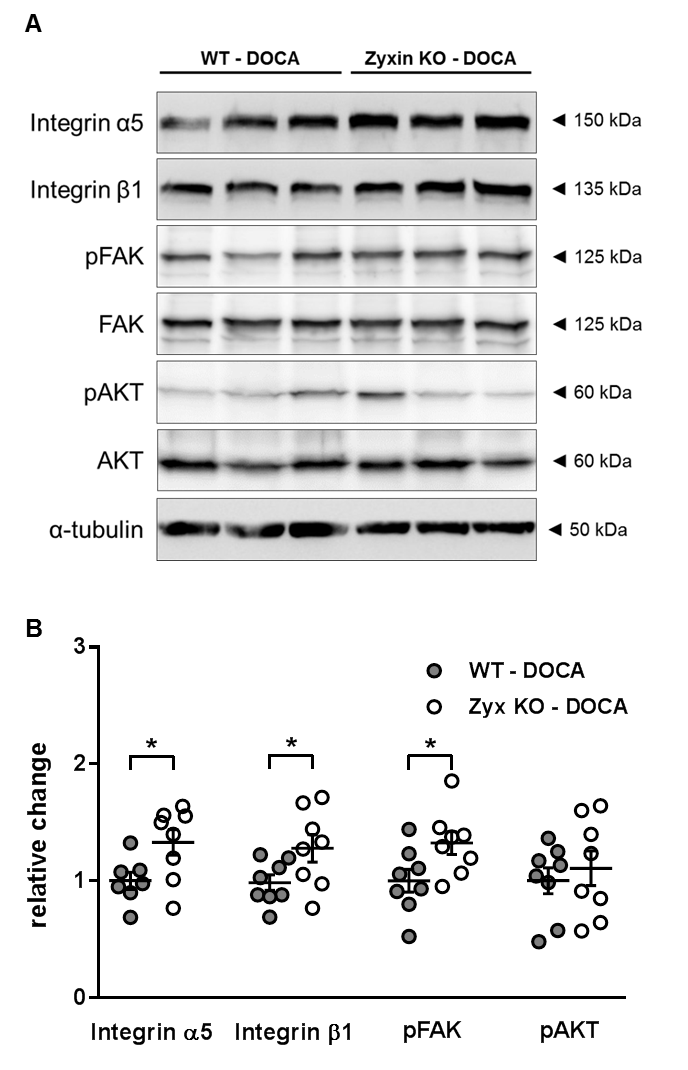


a

b

# Suppl. Fig. 7 Expression and activation of integrins in fibrotic hearts of 12-month old DOCA-salt treated mice.

a Representative Western blot analysis of cardiac tissue lysates from DOCA-salt treated WT and zyxin KO mice for integrin α5, integrin β1, focal adhesion kinase (FAK), phosphorylated focal adhesion kinase (pFAK), AKT, pAKT and α-tubulin. b Statistical summary of protein abundance in these hearts (*n=7-8)*, *p<0.05


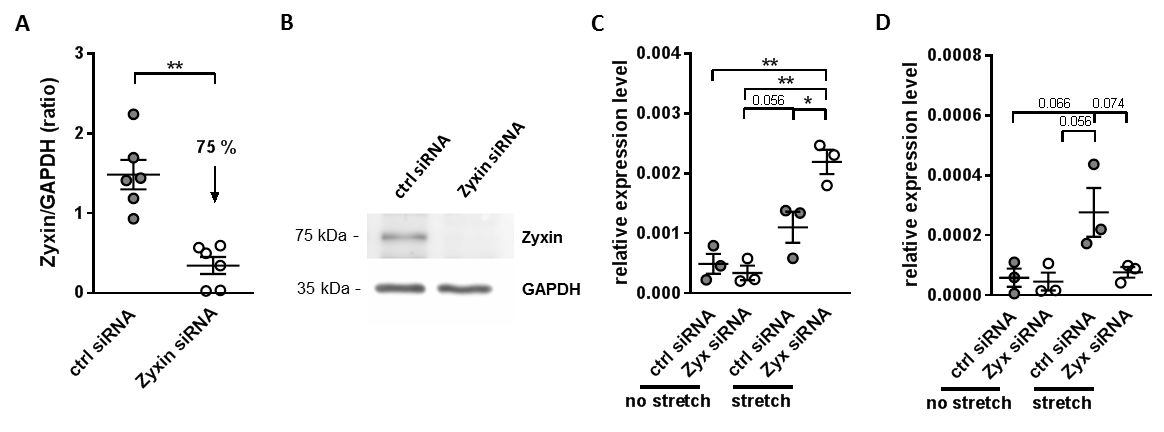


a

b

c

d

**relative Itga2 expression**

**relative Itgb6 expression**

Suppl. Fig. 8 Zyxin knock-down in microvascular endothelial cells.

a Knock-down efficiency of zyxin (Zyx) in microvascular endothelial cells (CI-muMEC) normalized to GAPDH (*n=6/6*). b Representative immunoblots of zyxin and GAPDH in CI-muMECs transfected with ctrl siRNA or zyxin siRNA. c, d *Itga2* (c) and *Itgb6 (d)* mRNA expression analyzed with an RT^2^-profiler array in control or zyxin knocked-down CI-muMECs cultured under static conditions or exposed to cyclic stretch (15% elongation at 0.5 Hz) for 6 hours (*n=3/3/3/3*). *p<0.05, **p<0.01

Suppl. Fig. 9 Resistivity index of 18 month old DOCA-salt treated mice.

The resistivity of the femoral arteries from these mice was analyzed via ultrasound measurements *in vivo* following 21 days of DOCA-salt treatment(*n=8*). *p<0.05

Suppl. Table 1: Outline of the age, genotype and treatment as well as the number of animals per group.

| age (months) | genotype | DOCA  (n) | Ang II  (n) | vehicle  (n) |
| --- | --- | --- | --- | --- |
| 6 | WT | 9 | 9 | 8 |
|  | zyxin KO | 12 | 10 | 8 |
| 12 | WT | 8 | 10 | 9 |
|  | zyxin KO | 8 | 9 | 7 |
| 18 | WT | 8 |  |  |
|  | zyxin KO | 8 |  |  |

Suppl. Table 2: Primer sequences and annealing temperatures used for quantitative real-time PCR analysis.

| gene | | primer sequence (5’ 🡒 3’) | annealing temperature | supplier |
| --- | --- | --- | --- | --- |
| *Rpl32* | forward | GGGAGCAACAAGAAAACCAA | 60 °C |  |
|  | reverse | ATTGTGGACCAGGAACTTGC |  |  |
| *Lox* | forward | CAGCCACATAGATCGCATGGT | 50 °C |  |
|  | reverse | GCCGTATCCAGGTCGGTTC |  |  |
| *Ctgf* | forward | CGAAGCTGACCTGGAGGAA | 58 °C |  |
|  | reverse | TTGGCGATTTTAGGTGTC |  |  |
| *Itgb1* | forward | TGGCAACAATGAAGCTATCGTG | 56 °C |  |
|  | reverse | GTAGGACAGTCTGGAGTCTCCACA |  |  |
| *Anp* |  | QT00250922 | 55 °C | Qiagen |
| *Bnp* |  | QT00107541 | 55 °C | Qiagen |

Suppl. Table 3: Conditions used for quantitative real-time PCR analysis.

| reaction step | temperature  (°C) | time  (s) | cycles  (n) |
| --- | --- | --- | --- |
| pre-denaturation | 95 | 300 |  |
| denaturation | 95 | 30 |  |
| annealing (primer specific) | 50-60 | 60 | 35-50 |
| elongation | 72 | 120 |  |
| final extension | 72 | 300 |  |
